# Dystonic opisthotonus: A “red flag” for neurodegeneration with brain iron accumulation syndromes?

**DOI:** 10.1002/mds.25490

**Published:** 2013-06-04

**Authors:** Maria Stamelou, Scarlett C Lai, Annu Aggarwal, Susanne A Schneider, Henry Houlden, Tu-Hsueh Yeh, Amit Batla, Chin-Song Lu, Mohit Bhatt, Kailash P Bhatia

**Affiliations:** 1 Sobell Department of Motor Neuroscience and Movement Disorders, University College London (UCL) Institute of NeurologyLondon, United Kingdom; 2 Division of Movement Disorders, Department of Neurology, Chang Gung Memorial Hospital at Linkou Medical Center and Chang Gung UniversityTaoyuan, Taiwan; 3 Neuroscience Research Center, Chang Gung Memorial Hospital at Linkou Medical CenterTaoyuan, Taiwan; 4 Center for Brain and Nervous Diseases, Kokilaben Dhirubhai Ambani Hospital and Medical Research InstituteMumbai, India; 5 Department of Neurology, University of KielKiel, Germany; 6 Department of Molecular Neuroscience, UCL Institute of NeurologyLondon, United Kingdom

**Keywords:** neurodegeneration with brain iron accumulation, NBIA, opisthotonus, retrocollis, extensor axial dystonia, *PLA2G6*, PANK2

## Abstract

Back arching was reported in one of the very first patients with neurodegeneration with brain iron accumulation syndrome (NBIAs) published in 1936. However, recent reports have mainly focused on the genetic and imaging aspects of these disorders, and the phenotypic characterization of the dystonia has been lost. In evaluating patients with NBIAs in our centers, we have observed that action-induced dystonic opisthotonus is a common and characteristic feature of NBIAs. Here, we present a case series of patients with NBIAs presenting this feature demonstrated by videos. We suggest that dystonic opisthotonus could be a useful “red flag” for clinicians to suspect NBIAs, and we discuss the differential diagnosis of this feature. This would be particularly useful in identifying patients with NBIAs and no iron accumulation as yet on brain imaging (for example, as in phospholipase A2, group IV (cytosolic, calcium-independent) [*PLA2G6*]-related disorders), and it has management implications. © 2013 The Authors. Movement Disorders published by Wiley Periodicals, Inc. on behalf of International Parkinson and Movement Disorder Society.

With the advent in genetics, a variety of complicated recessive dystonia syndromes have been identified, and the similarity in their clinical presentations makes the differential diagnosis for clinicians difficult.^1^,^2^ Hence, clinical clues and “red flags” may be an important help. Generally, in the differential diagnosis of dystonia, the phenomenology, the distribution combined with the age of onset, and the presence of other features are of great importance. For example, lower limb dystonia in an adult is a clue for secondary/heredo-degenerative dystonia rather than primary dystonia. Moreover, severe oromandibular dystonia points to certain disorders, such as neuroacanthocytosis, neuroleptic drug-induced dystonia, neurodegeneration with brain iron accumulation syndromes (NBIAs), or Lesch-Nyhan. Much less has been written about the diagnostic value and differential diagnosis of extensor truncal dystonia (or dystonic opisthotonus).

NBIAs cause complicated dystonia syndromes and are characterized by excessive iron deposition in the brain, particularly affecting the basal ganglia and mainly the globus pallidus. The 2 core NBIAs are the neuroaxonal dystrophies pantothenate kinase (PKAN)-associated and phospholipase A2, group IV (cytosolic, calcium-independent) (PLA2G6)-associated neurodegeneration (PLAN), whereas additional disorders recently have been described.^3^–^5^ In evaluating patients with NBIAs in our centers, we have observed typical action-induced dystonic opisthotonus, in which the trunk tends to arch backward when the patient stands and occasionally even when lying down and attempting to move. Here, we discuss the differential diagnosis of dystonic opisthotonus and the occurrence of this feature in NBIAs. We wish to highlight that dystonic opisthotonus may be a clinical clue that, together with other signs (such as oromandibular dystonia), should raise suspicion to test for these disorders.

## How Common is Dystonic Opisthotonus in NBIAs?

Numerous early and later case reports, in which clinical descriptions are detailed, describe neck arching and back arching in NBIAs^6^–^12^; in fact, back arching was noted in 1 of the very first described patients with this syndrome published in 1936 by Ludo von Bogaert.^6^,^7^ This confirms that dystonic opisthotonus may be a common feature of NBIAs. In most of those early described patients, opisthotonus tended to worsen with action and was observed mostly with young-onset forms^13^–^17^; this is not surprising, because generalized and/or truncal dystonia is more common in young-onset rather than atypical later-onset cases of NBIAs.^18^ Opisthotonus in neuroferritinopathy and aceruloplasminaemia, which typically have a later age of onset than PKAN and PLAN, has not been reported to the best of our knowledge. This may be related to the later age at onset and also to genetic factors.

A brief review of more recent literature on NBIAs reveals that, in large series of patients that were published mostly after the identification of causative genes, although generalized, truncal and neck dystonia are described, and a more specific phenotypic description of the dystonia is largely missing.^6^,^11^–^17^ For example, 87% of 52 patients with panthothenate kinase 2 (*PANK2*) mutations had dystonia, including action-induced axial dystonia, but it is unknown how many patients had extensor axial dystonia^19^; in the largest series of deep brain stimulation in 23 patients with NBIAs, the dystonia characteristics were not provided.^11^,^20^

**Figure 1:**
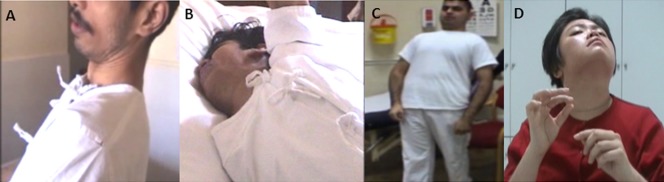
Photographs show (A) action-induced opisthotonus that occurs when sitting up from bed (Patient 1, *PANK2* mutations); (B) spontaneous, painful retrocollis and dystonic opisthotonus (Patient 2, *PANK2* mutations); (C) dystonic opisthotonus while walking (Patient 3, *PLA2G6* mutations); and (D) retrocollis (Patient 4, *PLA2G6* mutations).

We retrospectively evaluated all patients with NBIAs who have been seen at our center (London) the last 5 years (N = 8) and observed dystonic opisthotonus in 4 of 5 of patients with *PANK2* and *PLA2G6* mutations (see video segments 1–3), but not in patients with neuroferritinopathy or aceruloplasminaemia (N = 3), which is consistent with the literature, as mentioned above. Detailed clinical descriptions and imaging and genetic findings in these patients with opisthotonus have been published elsewhere but are summarized briefly in the video legend.^5^,^21^,^22^ There are some interesting aspects of these patients highlighted here. First, in most patients, the dystonic opisthotonus is action-induced, for example, when the patient stands up and starts walking (video segments 1, 3, and 4); (Figure 1) whereas, in Patient 2, this also may occur spontaneously while lying down. Second, Patients 3 and 4 did not have iron on brain magnetic resonance imaging (MRI) studies (including T2* sequences) or severe oromandibular dystonia, and the clinical clue to test for NBIAs, in fact, was the dystonic opisthotonus, which highlights the importance of this sign in the differential diagnosis. However, the true prevalence of dystonic opisthotonus in NBIAs needs to be assessed in larger case series.

## Differential Diagnosis of Dystonic Opisthotonus

The differential diagnosis of dystonic opisthotonus includes mainly secondary dystonias,^23^–^25^ while it is uncommon in primary dystonia.^26^ Classically, retrocollis has been described in tardive dystonia caused by use of dopamine receptor antagonists; approximately 36% to 50% of patients with tardive dystonia have retrocollis, and about half of these also have extensor truncal dystonia,^23^,^27^,^28^ which worsens during movement, especially walking.^23^,^27^–^29^

Because Wilson's disease is a common differential diagnosis in patients with young-onset dystonia syndromes, we screened 100 patients who had symptomatic neurologic Wilson's disease who were followed in the Wilson's disease clinic (Kokilaben Dhirubhai Ambani Hospital, Mumbai, India) for retrocollis and opisthotonus. We reviewed the medical records and serial videos that were taken at approximately 3-month intervals over the last 7 years. Consistent with other reports,^30^ axial symptoms related to dystonia were observed, and some patients presented with extensor truncal dystonia.

In patients diagnosed with dystonic cerebral palsy, opisthotonus has been described occasionally, but some patients with so-called cerebral palsy may have other conditions (including NBIAs)^7^,^33^–^36^; thus, “red flags” are important to avoid long delays in diagnosis.^31^–^34^ Opisthotonus also has been described in neurometabolic disorders (eg, glutaric aciduria, maple syrup urine disease, Lesch-Nyhan, dopa-responsive dystonias)^33^,^35^–^40^ (Table1). The very early age at onset, delayed motor milestones, truncal hypotonia, encephalopathic crisis, and intermittent painful dystonic posturing exacerbated by fever or infections are helpful clues to suspect a neurometabolic disease (see Table1).^39^,^41^ Conditions that reportedly cause back arching because of different etiologies, such as tetanus, strychnine poisoning, meningitis, and encephalitis, or “arc-de-cercle” in psychogenic dystonia are less likely to pose differential diagnostic problems with NBIAs.^35^,^42^–^46^

**Table 1:** The differential diagnosis of dystonic opisthotonus (incomplete list)

Differential diagnosis of dystonic opisthotonus	Further clues for the differential diagnosis
Drug-induced dystonia^23^,^28^,^53^	Often also retrocollis
	History of drug consume
NBIAs	Oromandibular dystonia
	Parkinsonism
	Iron accumulation in brain MRI
Glutaric aciduria^36^,^40^	Consanguinity
Maple syrup urine disease^35^,^37^,^38^	Perinatal history
	Very early age at onset
	Possibly delayed motor milestones
	Truncal hypotonia
	Encephalopathic crisis
	Intermittent painful dystonic posturing exacerbated by fever, infections
Wilson's disease	Kayser-Fleischer rings
	Oromandibular dystonia
	Wing-beating tremor
Lesch-Nyhan^54^	Oromandibular dystonia
	Self-injurious behavior
	Intellectual disability
Dopa-responsive dystonia (DYT5)^33^	Levodopa response
	Perinatal history
	Delayed motor milestones
Tyrosine hydroxylase deficiency	Oculogyric crisis
Aromatic L-amino acid decarboxylase deficiency	Perinatal history
	Delayed motor milestones
Sepiapterin reductase deficiency^55^	
Primary extensor truncal dystonia^26^	No further signs
Others (eg, meningitis, encephalitis, etc)	Dependent on the underlying cause

MRI, magnetic resonance imaging; NBIAs, neurodegeneration with brain iron accumulation syndromes.

## Which Is the Possible Pathophysiology?

The pathophysiologic explanation of the anatomic predilection for oromandibular and extensor truncal dystonia in these patients, as opposed to primary dystonias, remains unknown.^47^ However, the fact that these features also are present in patients with NBIAs in whom brain imaging does not show iron deposition implies that the clinical picture probably is not directly related to the iron but is related to the underlying neurodegeneration.^5^,^48^,^49^ This is supported by the fact that treatment with an iron-chelator, deferiprone, reduced iron in MRI studies but did not improve clinical symptoms.^49^ Moreover, the finding that dystonic opisthotonus responds to globus pallidus internus or subthalamic nucleus deep brain stimulation^11^,^15^,^50^ and, in some patients, also to levodopa^22^confirms that it is related to basal ganglia dysfunction as opposed to other conditions with nondystonic opisthotonus.

## Conclusion

We identified dystonic opisthotonus as a characteristic feature of NBIAs related to *PANK2* and *PLA2G6* mutations and suggest that this feature, together with other “red flags” for NBIAs (such as severe oromandibular dystonia) should raise suspicion to test for these disorders in patients with young-onset, complicated dystonia syndromes. Hence, these patients should have appropriate imaging, which includes T2* and susceptibility-weighted imaging to look for brain iron accumulation. Phenotypic “red flags” are important for clinicians for many reasons. First, some patients with NBIAs may not initially have evidence of iron accumulation in brain imaging (as in Patients 3 and 4 presented here), and suspicion for genetic testing can be mainly guided by phenotypic clues; otherwise, misdiagnosis for many years may occur.^31^,^51^ Second, the identification of these patients may have important management implications in view of current research on new treatment approaches.^49^,^52^ The true prevalence of this feature in NBIAs, along with other disorders described here, needs to be evaluated in larger studies.

## Legend to the Video

**Video 1.** Four patients with NBIAs and dystonic opisthotonus are shown. None of the patients were receiving neuroleptics before the onset of dystonic opisthotonus.

**Segment 1.** This Indian man aged 39 years carries homozygous mutations (c.1010A>C; p.Asp337Ala) in the *PANK2* gene. He developed decrease in visual acuity and dysarthria at age 12 years and lower limb dystonia and dystonic opisthotonus at age 14 years. On examination at age 34 years, he had reduced visual acuity, slow and hypometric vertical and horizontal saccades, generalized dystonia with prominent oromandibular dystonia, and severe dystonic opisthotonus, which was more evident while walking. Brain MRI revealed an “eye-of-the-tiger” sign.^21^

**Segment 2.** This Indian woman aged 36 years (the sister of patient 1) carries the same mutation. At age 13 years, she developed visual disturbances, dysarthia, and writer's cramp on the right.^21^ On examination at age 36 years, she was anarthric, and she had reduced visual acuity and pigmentary retinopathy with hypometric saccades; she also had generalized dystonia with oromandibular involvement, retrocollis, and dystonic opisthotonus with increased tone in all limbs. Brain MRI revealed an “eye-of-the-tiger” sign.

**Segment 3.** This Pakistani man aged 21 years, the product of double consanguinity, carries homozygous c.2239C_T (p.R747W) mutations in the *PLA2G6* gene.^5^ At age 18 years, he developed foot dystonia, cognitive decline, and personality changes. On examination at age 21 years, he had blepharoclonus, jerky saccadic pursuit, and asymmetric pyramidal features with spasticity, hyper-reflexia, and rigidity; bradykinesia; foot dystonia; and marked opisthotonus, which worsened with walking. Brain MRI revealed no iron deposition on T2* imaging.

**Segment 4.** This Taiwanese woman aged 25 years caries a compound heterozygous mutation of the *PLA2G6* gene (p.Asp331Tyr/p.Met358IlefsX). She noticed unsteady gait and easy falls at age 8 years, developed cognitive decline at age 18 years, and developed dystonia at age 22 years. Examination at age 25 years revealed retrocollis and dystonic opisthotonus induced by walking, parkinsonism, ataxic gait, intellectual impairment, and dysarthria. Brain MRI revealed cortical and cerebellar atrophy but no evidence of iron deposition on T2* sequences.^22^
